# INTERGENERATIONAL HEALTH PROMOTION PROJECT TO REDUCE OLDER ADULT SOCIAL ISOLATION AND LONELINESS: A FEASIBILITY STUDY

**DOI:** 10.1093/geroni/igae098.0705

**Published:** 2024-12-31

**Authors:** Jeremy Holloway

**Affiliations:** New Mexico Highlands University, Fargo, North Dakota, United States

## Abstract

Emerging research demonstrates that social isolation and loneliness are linked to significant physical and mental health conditions. To address these concerns, the Tellegacy program was developed as an intergenerational health-promoting intervention to ameliorate older adult social isolation and loneliness in an effort to increase wellness. The purpose of this study was to reflect on testing of the Tellegacy program as a behavioral intervention. University students trained in goal setting, mindfulness, and listening strategies were paired with 11 older adults in the northern Midwest area via weekly in-person and phone conversations. Oral reminiscence therapies were used and books containing their stories were given to the older adults after participation. Older adults were surveyed using the University of California Los Angeles Loneliness Scale, Satisfaction of Life Scale, and patient health questionnaire-9 (PHQ-9) scale to elucidate the effectiveness of the intervention. Improved scores in loneliness, satisfaction of life, and PHQ-9 demonstrated favorable improvements in older adults. Additional benefits for the student Legacy Builder were revealed from self-reported changes. This suggests the potential benefits of structured encounters between trained students and isolated or lonely older adults. The Tellegacy intergenerational feasibility program warrants further studies to fully demonstrate its impact on health outcomes.

